# Upregulating CXCR7 accelerates endothelial progenitor cell-mediated endothelial repair by activating Akt/Keap-1/Nrf2 signaling in diabetes mellitus

**DOI:** 10.1186/s13287-021-02324-7

**Published:** 2021-05-03

**Authors:** Chunyu Jiang, Ruiting Li, Chaoyang Xiu, Xu Ma, Hui Hu, Liming Wei, Yihan Tang, Mingyang Tao, Jungong Zhao

**Affiliations:** 1 Department of Interventional Therapy, Shanghai Ninth People’s Hospital, Shanghai Jiao Tong University of Medicine, No. 639 Zhi Zao Ju Road, Shanghai, 200233 People’s Republic of China; 2 Department of Radiology, The Sixth People’s Hospital, Affiliated to Shanghai Jiao Tong University, 600 Yi-Shan Road, Shanghai, 200233 People’s Republic of China; 3 Institute of Translational Medicine, Medical College, Yangzhou University, Yangzhou, 225001 People’s Republic of China

**Keywords:** Diabetes mellitus, Endothelial progenitor cells, CXCR7, Nrf2, Reendothelialization, Neointimal hyperplasia

## Abstract

**Background:**

Endothelial progenitor cell (EPC) dysfunction contributes to vascular disease in diabetes mellitus. However, the molecular mechanism underlying EPC dysfunction and its contribution to delayed reendothelialization in diabetes mellitus remain unclear. Our study aimed to illustrate the potential molecular mechanism underlying diabetic EPC dysfunction in vivo and in vitro. Furthermore, we assessed the effect of EPC transplantation on endothelial regeneration in diabetic rats.

**Methods:**

Late outgrowth EPCs were isolated from the bone marrow of rats for in vivo and in vitro studies. In vitro functional assays and Western blotting were conducted to reveal the association between C-X-C chemokine receptor type 7 (CXCR7) expression and diabetic EPC dysfunction. To confirm the association between cellular CXCR7 levels and EPC function, CXCR7 expression in EPCs was upregulated and downregulated via lentiviral transduction and RNA interference, respectively. Western blotting was used to reveal the potential molecular mechanism by which the Stromal-Derived Factor-1 (SDF-1)/CXCR7 axis regulates EPC function. To elucidate the role of the SDF-1/CXCR7 axis in EPC-mediated endothelial regeneration, a carotid artery injury model was established in diabetic rats. After the model was established, saline-treated, diabetic, normal, or CXCR7-primed EPCs were injected via the tail vein.

**Results:**

Diabetic EPC dysfunction was associated with decreased CXCR7 expression. Furthermore, EPC dysfunction was mimicked by knockdown of CXCR7 in normal EPCs. However, upregulating CXCR7 expression reversed the dysfunction of diabetic EPCs. The SDF-1/CXCR7 axis positively regulated EPC function by activating the AKT-associated Kelch-like ECH-associated protein 1 (keap-1)/nuclear factor erythroid 2-related factor 2 (Nrf2) axis, which was reversed by blockade of AKT and Nrf2. Transplantation of CXCR7-EPCs accelerated endothelial repair and attenuated neointimal hyperplasia in diabetes mellitus more significantly than transplantation of diabetic or normal EPCs. However, the therapeutic effect of CXCR7-EPC transplantation on endothelial regeneration was reversed by knockdown of Nrf2 expression.

**Conclusions:**

Dysfunction of diabetic EPCs is associated with decreased CXCR7 expression. Furthermore, the SDF-1/CXCR7 axis positively regulates EPC function by activating the AKT/keap-1/Nrf2 axis. CXCR7-primed EPCs might be useful for endothelial regeneration in diabetes-associated vascular disease.

## Background

Diabetes mellitus (DM) is a high-incidence, chronic, metabolic disease causing many complications, such as peripheral and cardiovascular disease, that severely influence patients’ quality of life [1, 2]. Interventional therapy, especially stent implantation, has been widely used for DM- associated vascular events and has significantly improved the prognosis of patients. However, unfavorable events after stent implantation, such as in-stent restenosis and late stent thrombosis, remain a serious clinical challenge [3, 4]. A large body of evidence has shown that endothelial injury caused by DM or interventional therapy contributes to these unfavorable vascular events [5–8]. Previous studies demonstrated that endothelial progenitor cells (EPCs) are crucial in endothelial repair [6–9]. However, DM impairs EPC function and decreases circulating EPC levels in both human patients and mice with diabetes [10–12], and these effects are associated with delayed reendothelialization [13, 14]. Thus, it is crucial to accelerate reendothelialization by understanding the potential mechanism underlying EPC dysfunction in DM.

CXC Chemokine Receptor 7 (CXCR7) is one of the most important ligand receptors for Stromal-Derived Factor-1 (SDF-1, also called CXCL12) and is expressed by EPCs [15–18]. The SDF-1/CXCR7 axis is involved in regulating the survival, adhesion, and angiogenesis of EPCs [15, 17, 18]. Moreover, dysfunctional EPCs derived from patients with hypertension have a decreased CXCR7 level, which is associated with delayed reendothelialization [19]. Dai et al. further confirmed that decreased CXCR7 expression was associated with dysfunction of late outgrowth EPCs in DM. Furthermore, elevated expression of CXCR7 enhances the resistance of EPCs to DM-induced oxidative damage and improves the therapeutic efficacy of EPCs in diabetic limb ischemia [18]. Collectively, these findings indicate that the delayed reendothelialization in DM may be associated with the impaired repair and adhesion capacities of EPCs, which are attributed to decreased CXCR7 expression. Furthermore, elevating CXCR7 expression in EPCs may accelerate reendothelialization by rescuing EPC dysfunction in DM. However, evidence supporting this hypothesis is lacking. Thus, late outgrowth EPCs were isolated to assess the association between CXCR7 expression and EPC dysfunction and determine the contribution of this mechanism to delayed reendothelialization in DM.

Few studies have reported the potential signaling mechanisms downstream of the SDF-1/CXCR7 axis involved in regulating the functional activity of EPCs. Nuclear factor erythroid 2-related factor 2 (Nrf2) is a critical redox sensor for oxidative stress [20, 21]. Activated Nrf2, released from Kelch-like ECH-associated protein 1 (Keap-1, the repressor protein of Nrf2), translocates to the nucleus; binds to antioxidant response elements (AREs); activates the transcription of its target antioxidant genes, including heme oxygenase-1 (HO-1) and NADPH quinone oxidoreductase-1 (NQO-1) [21, 22]; and counteracts EPC dysfunction caused by reactive oxygen species (ROS) [13, 23]. Thus, our study also assessed whether the SDF-1/CXCR7/AKT axis regulates EPC functional activity by activating the Keap-1/Nrf2 pathway.

## Methods and materials

### Study design

A single-blind randomized study was conducted to reveal the effect of EPCs on endothelial repair and the potential role of the CXCR7/AKT/keap-1/Nrf2 axis in regulating EPC functional activity. The reendothelialization rate at day 7 and 14 and *I*/*M* ratio at day 21 after EPCs transplantation were recognized as our primary outcome. Totally 125 rats were randomly divided into two groups: diabetic group and normal group. Then, the diabetic group was divided into six groups: control group, diabetic group, normal groups, CXCR7 group, CXCR7-EPCs^Nrf2-WT^ group, and CXCR7-EPCs^Nrf2-KD^ group. After groups and subgroups were divided, diabetic rat model was established and proved by rapid glucose meter. Then, EPCs were isolated from the bone marrow of normal or diabetic rats (10 rats each group) and used in subsequent in vitro and in vivo experiments. A carotid artery injury model was established in diabetic rats (totally, 105 rats) for subsequent in vitro study (the details are shown in supplement data 1). Finally, the results of the in vivo and in vitro studies were assessed by an analyst who was blinded to the experimental procedure.

### Diabetic rat model

Ten-week-old male Sprague-Dawley (SD) rats (200–250 g) were injected intraperitoneally with 55 mg/kg streptozotocin (STZ; Sigma-Aldrich, St. Louis, USA). On days 7 and 14, rats with fasting blood glucose levels higher than 14 mmol/L were considered diabetic and included in subsequent experiments. If fasting blood glucose levels were lower than 14 mmol/L, another STZ injection was conducted to assure that diabetic rat model was constructed successfully.

### EPCs isolation and culture

EPCs were isolated from the bone marrow of normal rats and rats with diabetes. The isolation, culture, and identification of EPCs were conducted as described in a previous study [23]. In brief, bone marrow was isolated from the femur and tibia and subjected to digestion, grinding, filtration, and resuspension in 10 mL of phosphate-buffered saline (PBS). EPCs were isolated from the cell suspension by Ficoll gradient centrifugation (1500×*g*) for 10 min at room temperature and cultured in endothelial basal medium (Lonza Group Ltd.) containing growth factors, based on the manufacturer’s instructions.

Fluorescent staining, immunocytochemistry, and flow cytometry were conducted to reveal the characteristics of the EPCs used in our study. In brief, fluorescent staining was used to detect the uptake of DiI-conjugated acetylated low-density lipoprotein (ac-LDL) (DiI-ac-LDL; Molecular Probes; Thermo Fisher Scientific, Inc.) and binding of FITC-UEA-l (Sigma-Aldrich; Merck KGaA). For immunocytochemistry, cells were fixed, incubated with primary antibodies overnight, and then incubated with secondary antibodies for 1 h. The cells were then washed three times and visualized using a fluorescence microscope (Leica AF6000). Antibodies specific for CD31 (1:100, Cat No. ab222783), CD34 (1:100, Cat No. ab81289), and vWF (1:100, Cat No. ab216566) were obtained from Abcam (Cambridge, Cambs., UK). For flow cytometry, cells were fixed and were then incubated with the following primary antibodies: BB515-conjugated mouse anti-human CD31 (Cat No. 565408), APC-conjugated mouse anti-human CD34 (Cat No. 560940), PE-conjugated mouse anti-human VEGFR-2 (Cat No. 560872), and FITC-conjugated mouse anti-human CD45 (Cat No. 554883). The antibodies were obtained from BD Biosciences (San Jose, CA, USA). Nonspecific fluorescence was assessed by incubation of similar cell aliquots with isotype-matched mouse monoclonal antibodies. Cells were washed with PBS and analyzed by using a GuavaeasyCyte™ Flow Cytometer (Millipore, Billerica, MA, USA).

### Lentiviral transduction and RNA interference

Lentiviral transduction and RNA interference were conducted to upregulate or downregulate, respectively, the expression of CXCR7 to confirm the association between CXCR7 expression and EPC dysfunction.

Recombinant lentivirus encoding CXCR7 was constructed using the pLVX-EGFP-3FLAG-Puro vector (Shanghai Sunbio Medical Biotechnology, Shanghai, China). In brief, EPCs were seeded into 24-well plates at a density of 1 × 10^5^ cells/well and incubated overnight. EPCs were then transduced overnight with purified lentiviral vectors that expressed recombinant CXCR7 at a multiplicity of infection of 25 in the presence of 3 μg/mL polybrene (Sigma, MO, USA). After 24 h of infection, the medium was replaced with 2 mL of fresh medium. The lentiviral transduction efficiency was determined by assessing lentiviral expression of green fluorescent protein (GFP). Apparent GFP expression was observed 48 h after transduction and peaked 72 h after transduction. The levels of CXCR7 expression were confirmed by Western blot analysis.

RNA interference was conducted to downregulate the expression of CXCR7 in EPCs. The sequences of the RNA interference and negative control (NC) constructs were as follows: CXCR7 siRNA sense, 5′-GGAAGAUCAUCUUCUCCUATT-3′ and antisense, 5′-UAGGAGAAGAUGAUCUUCCGG-3′; NC, 5′-UUCUCCGAACGUGUCACGUTT-3′; antisense, 5′-ACGUGACACGUUCGGAGAATT-3′. Transfection was performed as described previously [24]. In brief, siRNA transfection was performed using Lipofectamine 2000 reagent (Invitrogen, Carlsbad, CA, USA) following the manufacturer’s protocols. EPCs were plated into 6-well plates at a density of 5 × 10^5^ cells/well and incubated overnight. The diluted siRNA constructs and Lipofectamine 2000 reagent were mixed at a ratio of 1:1 (4 pmol siRNA to 4 μL of Lipofectamine 2000) and incubated for 20 min at room temperature. Finally, 400 μL of the mixture was added to each well to achieve a final volume of 2 mL, and the cells were continuously incubated for 48 h before starting subsequent experiments.

Nrf2 expression in CXCR7-primed EPCs was knocked down to reveal the detailed mechanism underlying the regulation of EPC functional activity by the SDF-1/CXCR7 axis. In brief, EPCs were infected with lentiviruses containing shRNA against Nrf2 or nonsense shRNA (Genomeditech, Shanghai, China). The sequences of the Nrf2 and nonsense shRNA constructs are as follows:

Nrf2-shRNA 1, 5′- GGGTAAGTCGAGAAGTGTTTG -3′

Nrf2-shRNA 2, 5′- GGACCTAAAGCACAGCCAACA -3′

Nrf2-shRNA 3, 5′- GCAAGAAGCCAGATACAAAGA -3′

Nonsense shRNA, 5′- TTCTCCGAACGTGTCACGTAG -3′

Transfection was performed following a previously described protocol. After transfection for 48 h, the expression of Nrf2 was determined by Western blot analysis. Then, EPCs were infected with lentiviruses containing the shRNA against Nrf2 with the highest knockdown efficiency (lowest expression of Nrf2) or with nonsense shRNA determined by western blot analysis.

### EPC adhesion assay

The adhesion of EPCs to human umbilical vein endothelial cells (HUVECs, Cell Resource Center of Shanghai Institute for Biological Sciences, Chinese Academy of Sciences; Cat No. 3131C0001000200023) was assessed by plating these cells in 24-well plates. First, HUVECs were plated in 24-well plates to form a monolayer. Then, the nonattached cells were washed away with PBS. DAPI (10 μg/mL) was used for staining HUVECs. EPCs were cultured in medium containing DiI dye (4 mg/mL) for 30 min at 37 °C following the manufacturer’s protocol. DiI-labeled EPCs were digested and harvested after three washes with PBS. DiI-labeled EPCs were then added to the plate and were then incubated with the HUVEC monolayer for 2 h, after which nonattached EPCs were washed away with PBS. Adherent EPCs were counted in five random fields under an Olympus microscope at × 400 magnification.

### Repair capacity of EPCs in vitro

The repair capacity of EPCs in vitro was assessed using a scratch assay. In brief, EPCs were plated in 96-well plates to form a monolayer after 48 h of culture. Then, the confluent monolayer was scratched using a p200 pipette tip (1 mL) containing serum-free medium. The wound area was imaged under a microscope (magnification, × 100; Olympus Corporation) at baseline (0 h) and after 24 h, and the data were then analyzed using ImageJ software (National Institutes of Health).

### Western blot analysis

EPCs were incubated for 24 h before protein extraction with a protein extraction kit (Solarbio, Beijing, China) and quantification with a bicinchoninic acid protein assay kit (Solarbio). Protein extracts were subjected to SDS-PAGE (KeyGEN, Nanjing, China) followed by transfer onto polyvinylidene fluoride membranes (Roche, IN, USA). The following primary antibodies (all obtained from Abcam (Life Technologies, CA, USA)) were used: anti-CXCR7 (1:1500, Cat No. ab138509), anti-GAPDH (1:10000, Cat No. ab8245), anti-p-Akt (1:5000, Cat No. ab38449), anti-Keap-1 (1:1000, Cat No. ab139729), anti-Nrf2 (1:1000, Cat No. ab92946), anti-HO-1 (1:2000, Cat No. ab189491), and anti-NQO-1 (1:1000, Cat No. ab80588). Subsequently, the membranes were incubated with secondary antibodies (1:1000, Cat No. bs-0346R-HRP; Beijing Boaosen Biotechnology Co., Ltd.) for 2 h at room temperature. Protein bands were visualized using an Epson Photo 1650 scanner (Seiko Epson Corp., Japan).

### Rat model and treatment regimens

The vascular injury rat model was used to assess the effect of putative EPCs on intimal repair. After establishment of the rat model of type I diabetes described above, the vascular injury model was established as described previously [23]. In brief, the vascular endothelium was injured by a guidewire as used in percutaneous transluminal angioplasty. The operation was performed under anesthesia with pentobarbital sodium. Then, the bifurcation of the left carotid artery was exposed, and the common, internal, and external carotid arteries were separated to temporarily restrict blood flow. The common carotid artery was denuded by three repeated passages of the 0.38-mm flexible angioplasty guidewire through the external carotid artery. The denuded segment encompassed a total length of 5 mm from the carotid bifurcation. The external carotid artery was permanently ligated after the guidewire was removed, and the temporary ligatures were released to allow restoration of blood flow followed by skin suture.

Then, 1.5 mL of saline (control group) or a cell suspension (1 × 10^6^ cells/mL) of normal EPCs (derived from normal rats; normal group), diabetic EPCs (derived from rats with diabetes; diabetic group), CXCR7-EPCs (derived from rats with diabetes, CXCR7-EPC group), CXCR7-EPCs^Nrf2-WT^ (derived from normal rats; CXCR7-EPC^Nrf2-WT^ group), or CXCR7-EPCs^Nrf2-KD^ (derived from normal rats; CXCR7-EPC^Nrf2-KD^ group) was injected into the circulation via the tail vein immediately and 12 h after establishment of the vascular injury model.

### Assessment of reendothelialization and neointimal hyperplasia

The reendothelialization rate was assessed by Evans blue staining on days 7 and 14 after treatment. In brief, 0.5 mL of 0.5% Evans blue dye was injected intravenously via the tail vein 30 min before sacrifice. Cardiac perfusion was then performed via the bilateral jugular vein with formaldehyde fixation for 5 min followed by washing with PBS until the effluent ran clear. The common carotid artery was harvested 4 mm from the bifurcation after measurement by a Vernier caliper and opened longitudinally using microscissors. Then, an image of the vessel was acquired with a stereomicroscope (DVM6, Leica). Digital images were analyzed using NIH ImageJ 1.63 software. The total endothelial area in all groups was first measured and analyzed. After confirming that the total endothelial area did not differ significantly among the groups, the Evans blue-stained and unstained areas were measured to calculate the reendothelialization rate (unstained area/total area).

Neointimal hyperplasia was assessed using hematoxylin and eosin (HE) staining and Masson trichrome staining on day 21 after treatment. Denuded arteries were harvested from rats and immersed in formalin for 24 h. The neointimal thickness was assessed using the intimal area-to-medial area ratio (*I*/*M*) in HE-stained axial sections. A pathologist blinded to the treatment regimen assessed all specimens using NIH ImageJ 1.63 software.

### Statistical analysis

Data are expressed as the mean ± standard deviation values. One-way or multi-way analysis of variance (ANOVA) with post hoc LSD comparisons with polynomial contrasts was used to determine significant differences between pairs of subgroups at each time point. *p* values < 0.05 were considered to be statistically significant. SPSS 20.0 software (IBM Corp., Armonk, NY, USA) was used to perform statistical analysis.

## Results

### Characteristics of EPCs

**Fig. 1:**
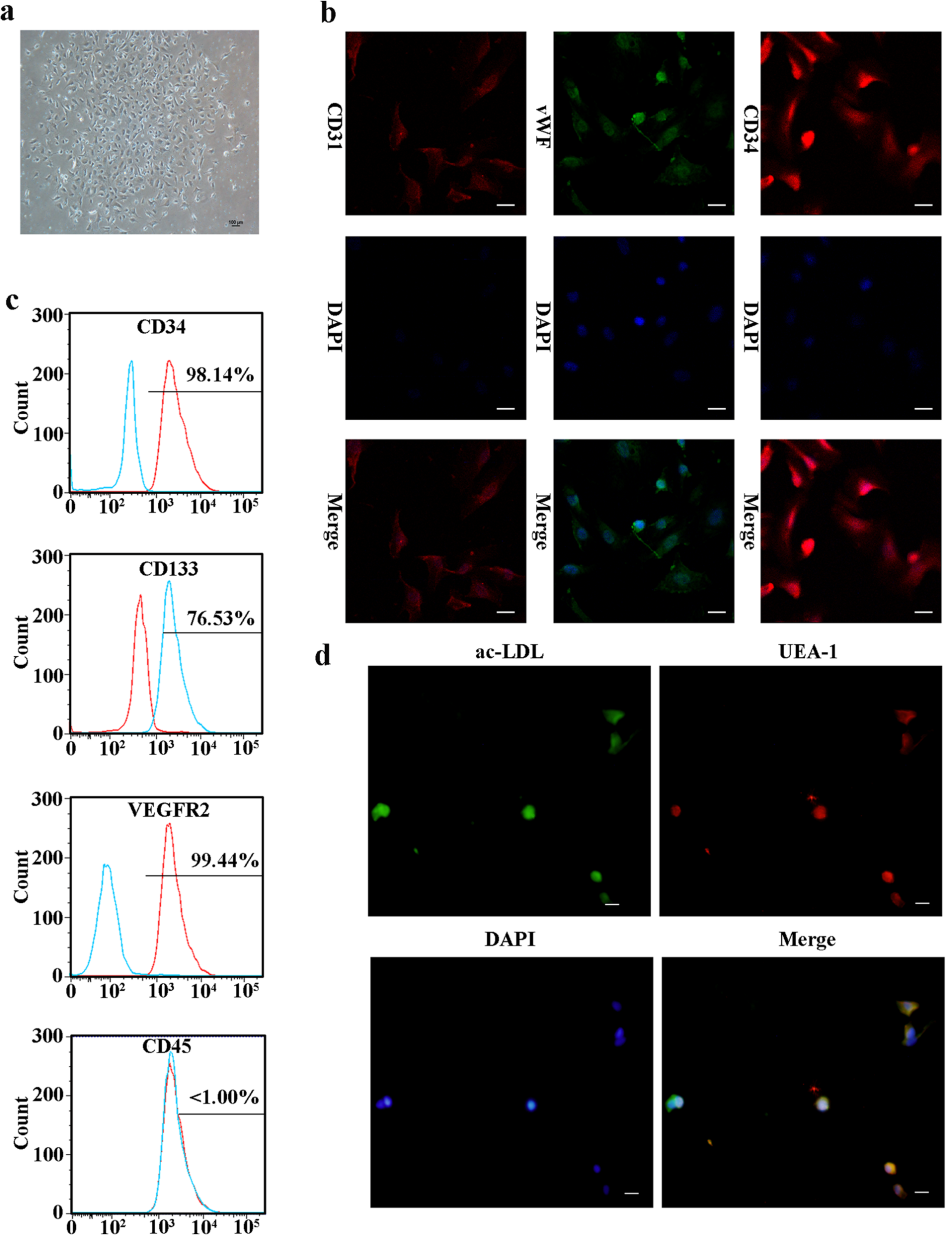
EPC characteristics. **a** EPCs showed a typical endothelial-like cobblestone morphology after culture for 21 days. **b** Immunofluorescence staining showed that EPCs were positive for v-WF, CD34, and CD31 staining (bar = 150 μm). **c** Flow cytometric analysis showed that EPCs were positive for CD34, VEGFR2, and CD133 but negative for CD45. **d** EPCs could take up ac-LDL and bind to UEA-1 (bar = 100 μm)

EPC clusters appeared 21 days after culture initiation (Fig. 1a). Three markers (v-WF, CD34, and CD31) were used for immunofluorescence detection to validate the harvested EPCs (Fig. 1b). Flow cytometric analysis showed that the EPCs were positive for CD34, VEGFR2, and CD133 but negative for CD45 (Fig. 1c). In addition, these cells showed positive staining for ac-LDL and the endothelial-specific lectin UEA-1 (Fig. 1d), further confirming their identity as late outgrowth EPCs (LOCs).

### DM downregulated CXCR7 expression and attenuated the functional activity of EPCs

**Fig. 2:**
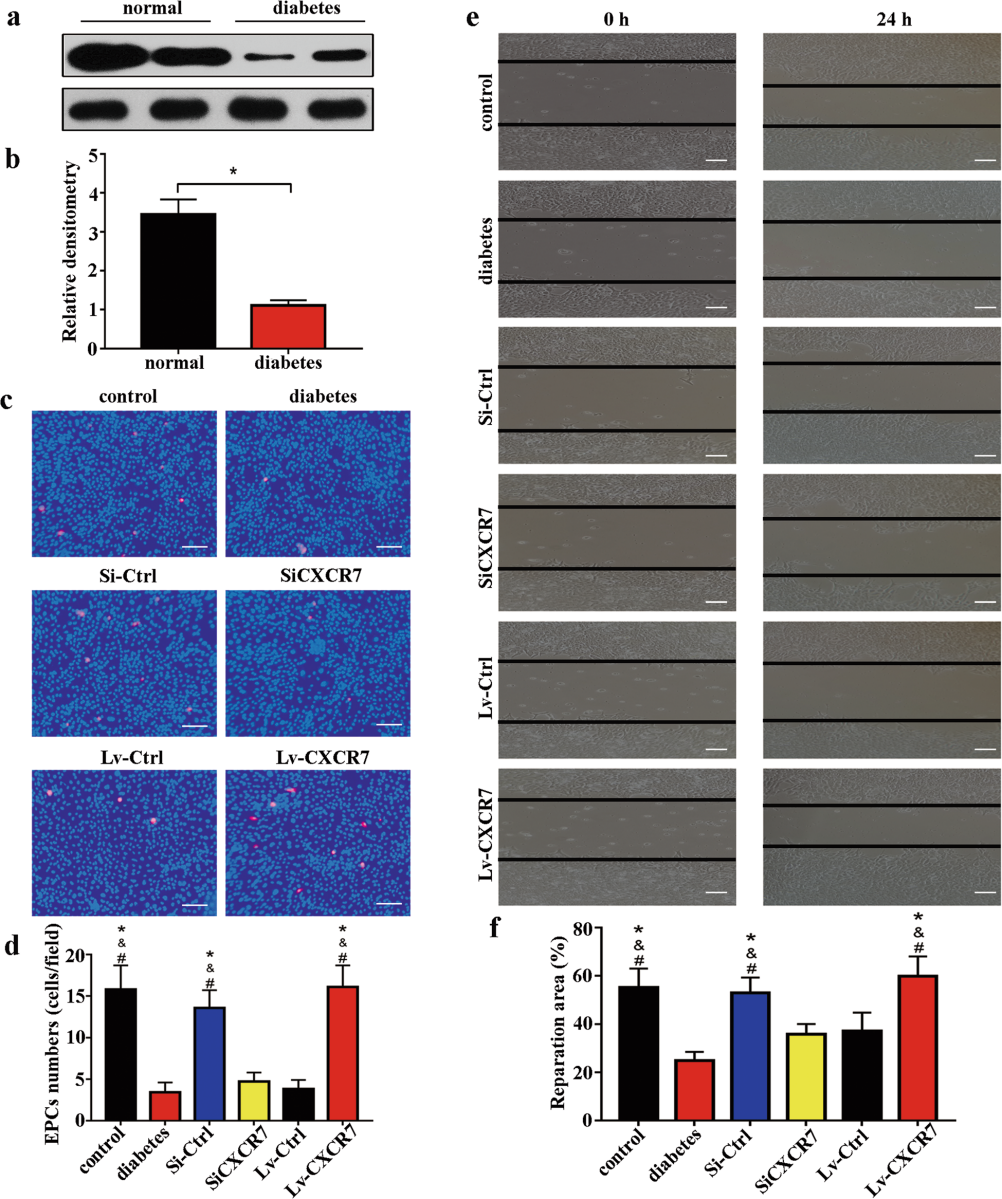
Elevating CXCR7 expression enhanced the functional activity of EPCs. **a**, **b** CXCR7 expression in diabetic EPCs (diabetic group) and normal EPCs (normal group) (*n* = 5). **c**, **d** Capacity of EPCs in different groups to adhere to HUVECs (bar = 100 μm; *n* = 5). **e**, **f** Repair capacity of EPCs in different groups (bar = 200 μm; *n* = 5) (*p < 0.05, compared with group diabetes; ^&^*p* < 0.05, compared with group SiCXCR7; ^#^*p* < 0.05, compared with group Lv-Ctrl)

Late outgrowth EPCs were isolated from the bone marrow of normal rats and rats with diabetes to determine the effect of DM on CXCR7 expression and the functional activity of EPCs. The expression of CXCR7 was obviously decreased in EPCs derived from rats with diabetes compared with EPCs from normal rats (Fig. 2a, b). In addition, the adhesion capacity of diabetic EPCs was attenuated compared with that of normal EPCs (normal group vs diabetic group: 23.8 ± 4.9 vs 9.4 ± 1.2 cells/field, *p* = 0.014) (Fig. 2c, d). Furthermore, the scratch assay showed that the in vitro repair capacity of EPCs was attenuated in the context of DM (normal group vs diabetic group, 52.83 ± 7.81% vs 24.93 ± 3.69%, *p* = 0.038; Fig. 2e, f).

To confirm the association between CXCR7 expression and EPC dysfunction, gain- and loss-of-function studies were performed by siRNA-mediated knockdown in EPCs obtained from normal rats and lentivirus-mediated overexpression in EPCs obtained from rats with diabetes. The studies showed that EPC dysfunction was mimicked by knockdown of CXCR7. Downregulating CXCR7 expression impaired the adhesion (normal group vs SiCXCR7 group, 16.8 ± 3.9 vs 4.6 ± 1.1 cells/field, *p* = 0.017) and repair (normal group vs SiCXCR7 group, 52.83 ± 7.81% vs 35.31 ± 4.27%, *p* = 0.041) capacities of normal EPCs. In contrast, increasing CXCR7 expression significantly reduced the dysfunction of diabetic EPCs, including restoring the adhesion (diabetic group vs Lv-CXCR7 group, 3.9 ± 1.2 vs 17.1 ± 3.2 cells/field, *p* = 0.01) and repair (diabetic group vs Lv-CXCR7 group, 24.93 ± 3.69% vs 67.5 ± 8.49%, *p* = 0.026) capacities in vitro (Fig. 2c–f). Collectively, these results indicated that the dysfunction of diabetic EPCs was attributed to the decrease in CXCR7 expression.

### Upregulating CXCR7 accelerated endothelial repair by EPCs and attenuated neointimal hyperplasia in DM

**Fig. 3:**
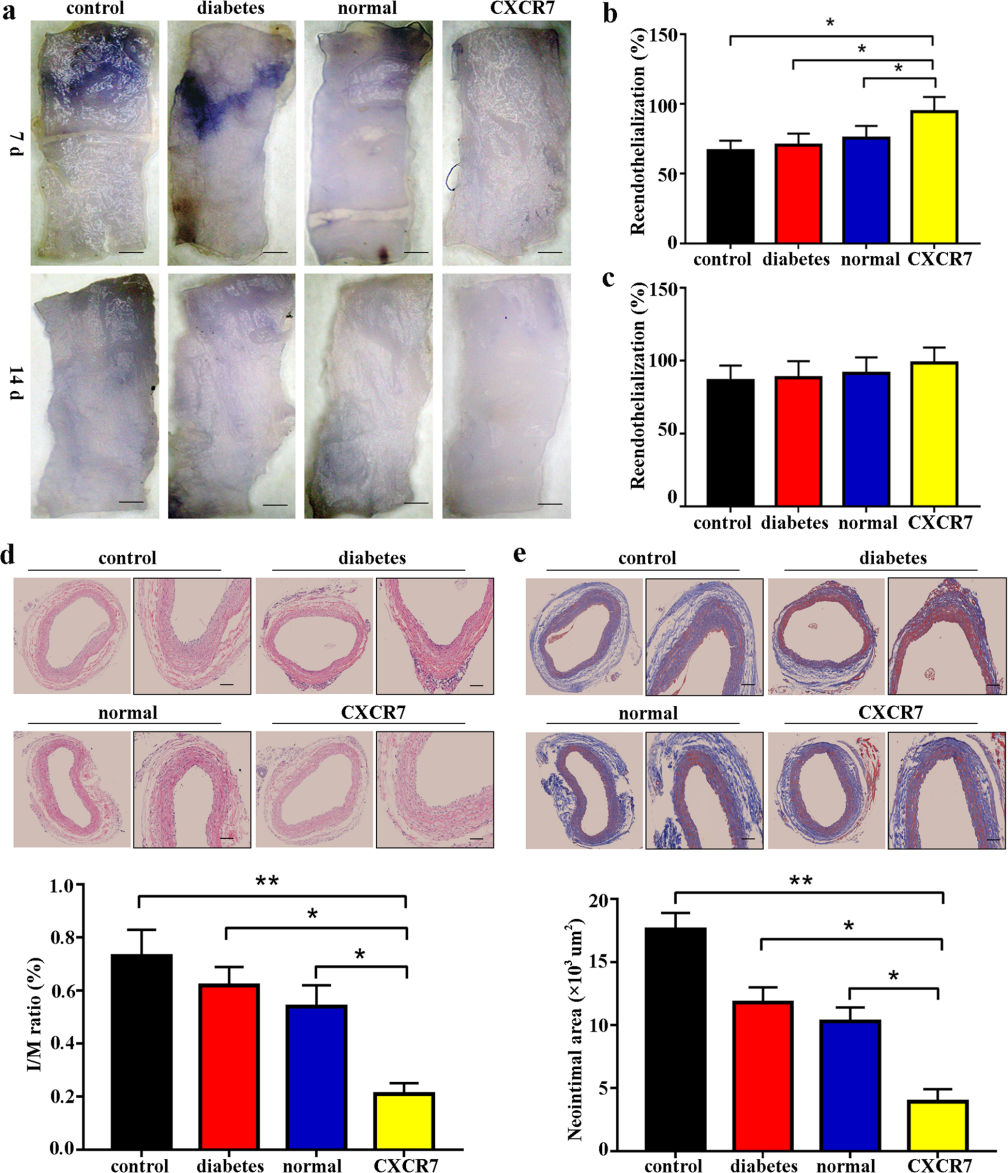
Upregulating CXCR7 expression accelerated reendothelialization and inhibited neointimal hyperplasia. **a** Representative images of Evans blue staining (bar = 500 μm; *n* = 5 per group). **b** Reendothelialization rate in different groups after 7 days. **c** Reendothelialization rate after 14 days. **d** *I*/*M* ratio of HE-stained EPCs 21 days after treatment (bar = 150 μm; *n* = 5 per group). **e** Neointimal area assessed by Masson trichrome staining 21 days after treatment (bar = 150 μm; *n* = 5 per group) (*p < 0.05; **p < 0.001)

Saline-treated EPCs (control group), normal EPCs (normal group), diabetic EPCs (diabetic group), and CXCR7-primed EPCs (CXCR7 group) were transplanted into rats with diabetes after induction of vascular injury to determine whether the elevated CXCR7 level accelerated EPC-mediated endothelial repair. The reendothelialization rate, *I*/*M* ratio, and neointimal area were used to assess endothelial regeneration and neointimal formation in the different groups. CXCR7-EPC transplantation effectively accelerated reendothelialization on day 7 (control group vs CXCR7 group, 66.41 ± 7.38% vs 94.66 ± 9.6%, *p* = 0.0298) but not on day 14 (control group vs CXCR7 group, 86.46 ± 10.35% vs 98.56 ± 10.67%, *p* = 0.398) after vascular injury in the DM model (Fig. 3a–c). Furthermore, CXCR7-EPC transplantation significantly reduced neointimal formation at 21 days after treatment (control group vs CXCR7 group: *I*/*M* ratio, 75.63 ± 10.45% vs 23.85 ± 5.67%, *p* < 0.001; neointimal area, 17.75 ± 2.19 vs 4.72 ± 1.33, *p* < 0.001). However, transplantation of normal or diabetic EPCs failed to accelerate endothelial repair and attenuate neointimal hyperplasia in the DM model (Fig. 3d, e). Collectively, these findings indicated that upregulating CXCR7 expression reversed diabetic EPC dysfunction and accelerated EPC-mediated endothelial repair in DM.

### Elevation of CXCR7 stimulated HO-1 and NQO-1 expression by activating the Akt-associated Keap-1/Nrf2 axis

The underlying mechanism by which the SDF-1/CXCR7 axis regulates EPC function remains largely unknown. Previous studies showed that the Keap-1/Nrf2 axis was crucial in positively regulating EPC function by activating target antioxidant genes. Thus, the SDF-1/CXCR7 axis was hypothesized to positively regulate EPC function by activating the Akt-associated Keap-1/Nrf2 axis. To confirm this hypothesis, EPCs were obtained from the bone marrow of wild-type rats, and the protein levels of relevant signaling molecules, including p-Akt, Keap-1, Nrf2, HO-1, and NQO-1, were assessed by Western blot analysis.

**Fig. 4:**
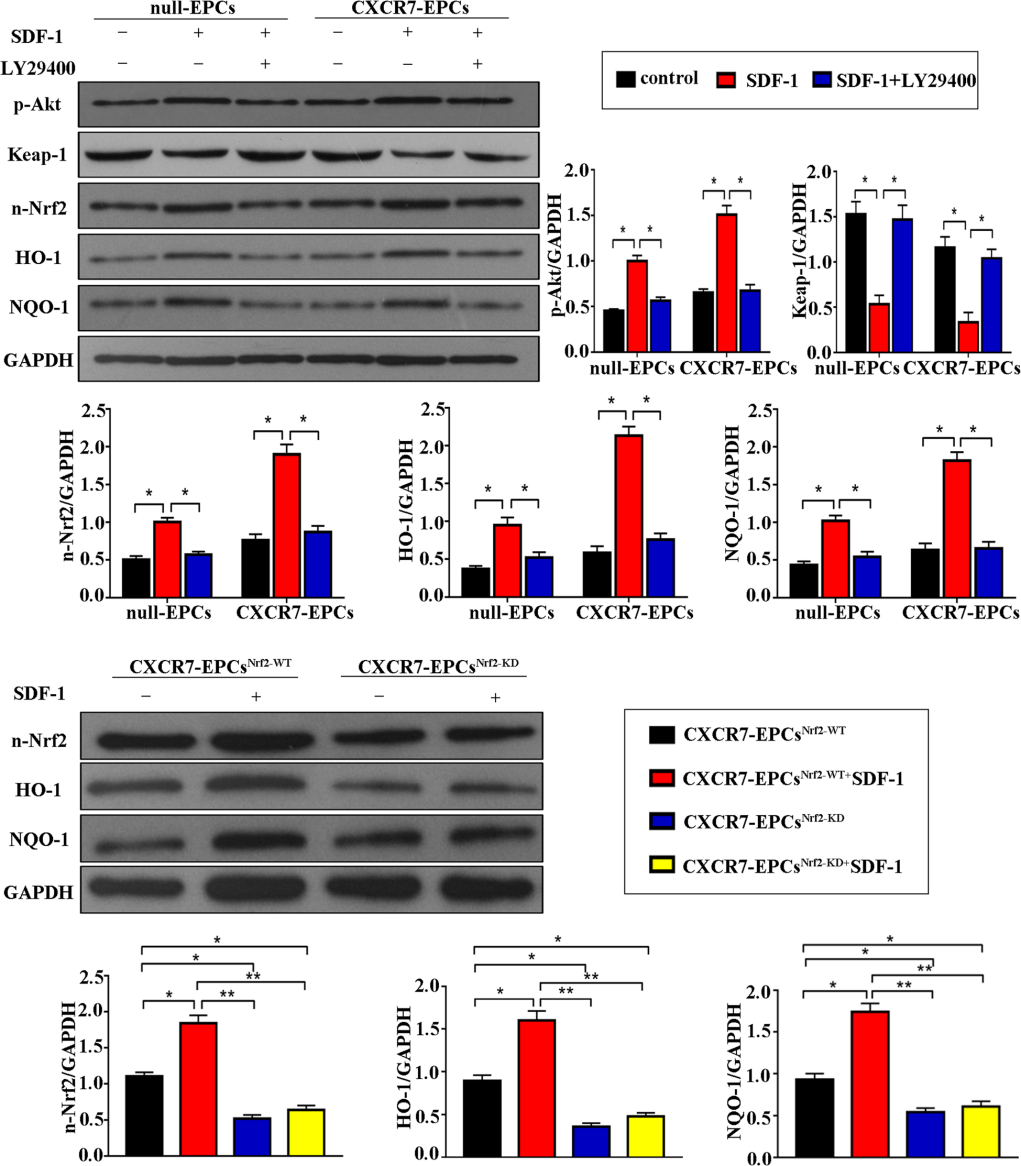
Upregulating CXCR7 expression activated the Akt-associated Keap-1/Nrf2 axis. **a** Levels of p-Akt, Keap-1, n-Nrf2, HO-1, and NQO-1. **b** Expression of HO-1 and NQO-1 after blockade of Nrf2 (*p < 0.05; **p < 0.001)

The p-Akt level in CXCR7-EPCs was higher than that in normal EPCs after pretreatment with SDF-1. Moreover, pretreatment with SDF-1 significantly decreased the Keap-1 level but promoted the accumulation of nuclear Nrf-2 in CXCR7-EPCs compared with normal EPCs. The HO-1 and NOQ-1 levels were also higher in CXCR7-EPCs than in normal EPCs (Fig. 4a). Then, Akt was blocked with LY29400 in normal EPCs and CXCR7-EPCs. Blockade of Akt inactivated the Keap-1/Nrf2 axis and decreased the expression of its downstream target genes in both normal and CXCR7-EPCs (Fig. 4a). Collectively, these results indicated that upregulation of CXCR7 controlled EPC functional activity by activating the Akt-associated Keap-1/Nrf2 axis.

### Knockdown of Nrf2 attenuated the functional activity and endothelial repair capacity of CXCR7-EPCs

**Fig. 5:**
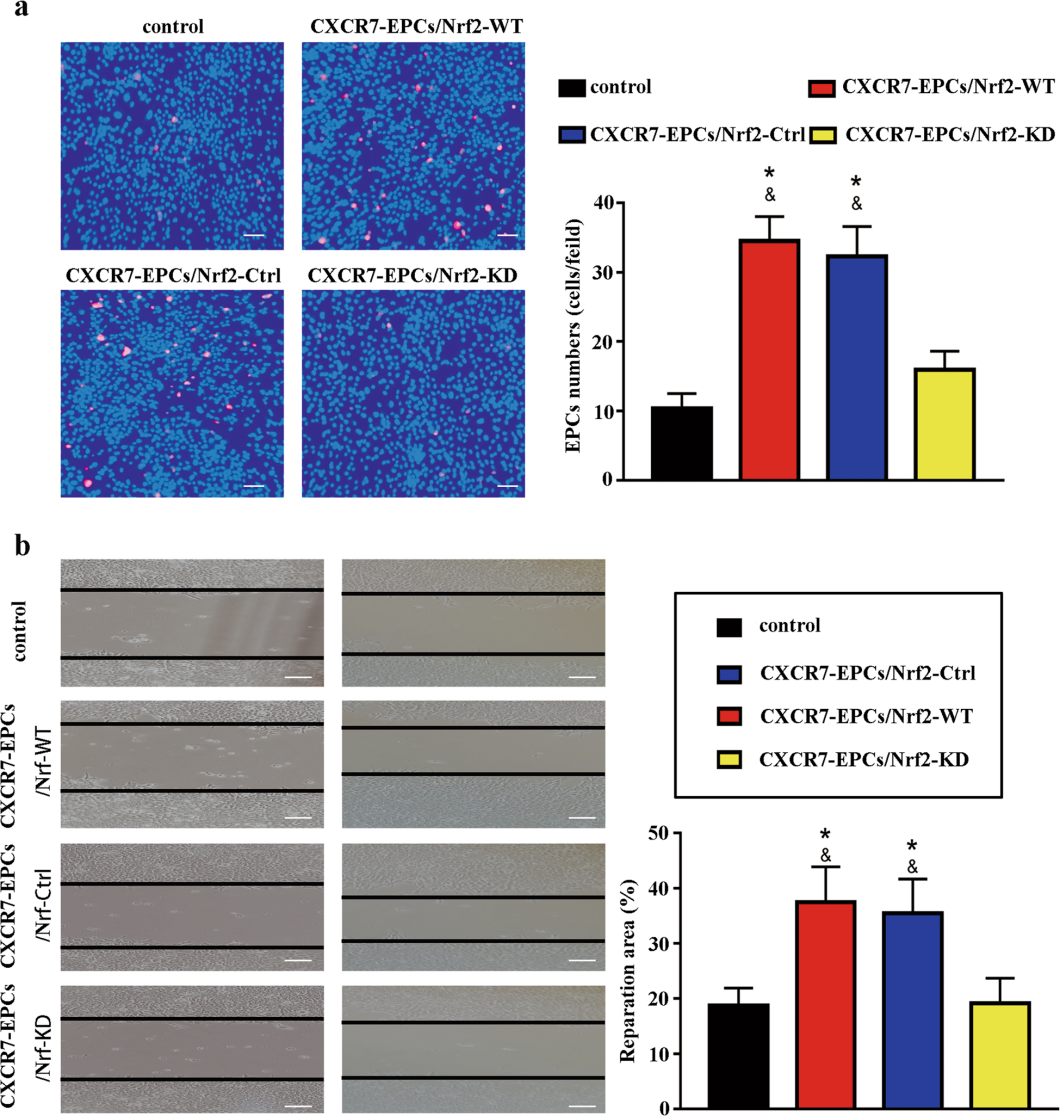
Knockdown of Nrf2 attenuated the functional activity of CXCR7-EPCs. **a** Differences in adhesion capacity among all groups (bar = 100 μm; *n* = 5). **b** Differences in repair capacity among all groups (bar = 200 μm; *n* = 5) (*p < 0.05, compared with control group; ^&^*p* < 0.05, compared with group CXCR7-EPC^Nrf2-KD^ group)

Nrf2 expression was knocked down with shRNA to further confirm the association between the Keap-1/Nrf2 axis and the SDF-1/CXCR7 pathway. Knockdown of Nrf2 expression significantly decreased the Nrf2 level, resulting in reductions in HO-1 and NOQ-1 levels in both normal EPCs and CXCR7-EPCs (Fig. 4b). Moreover, knockdown of Nrf2 abolished the protective effects of CXCR7 on EPC functional activity in vitro (Fig. 5). Both the adhesion capacity (CXCR7-EPC^Nrf2-KD^ group vs CXCR7-EPC^Nrf2-WT^ group, 15.94 ± 2.78 vs 34.5 ± 3.28 cells/field, *p* = 0.036) and the repair capacity (CXCR7-EPC^Nrf2-KD^ group vs CXCR7-EPC^Nrf2-WT^ group, 19.17 ± 4.69% vs 37.54 ± 6.42%, *p* = 0.029) were significantly attenuated in CXCR7-EPCs after blockade of Nrf2 (Fig. 5).

**Fig. 6:**
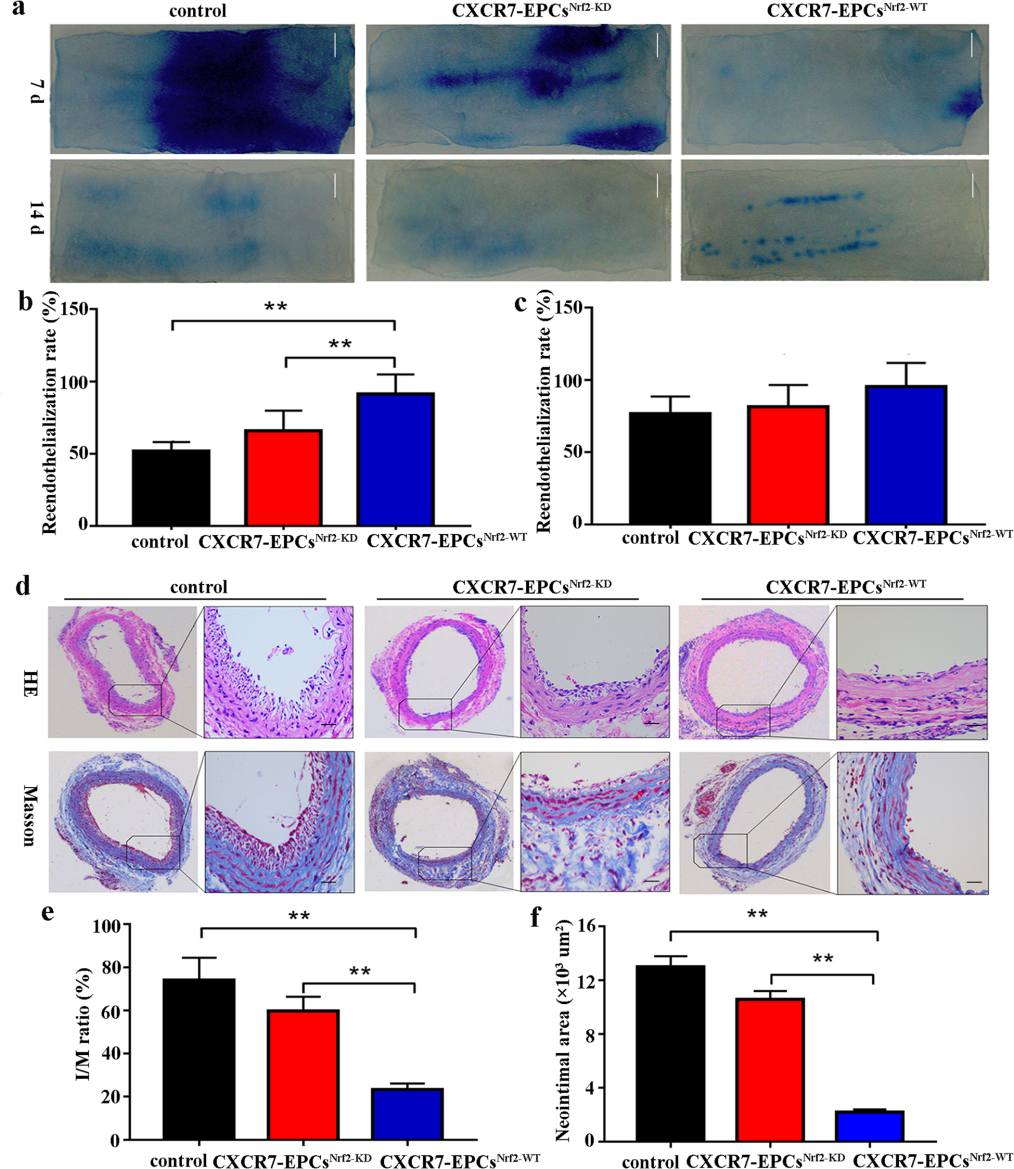
Knockdown of Nrf2 impaired endothelial repair of CXCR7-EPCs in vivo. **a** Representative images of Evans blue staining after EPC transplantation (bar = 500 μm; *n* = 5 per group). **b** Reendothelialization rate in different groups after 7 days. **c** Reendothelialization rate in different groups after 14 days. **d** Representative images of HE and Masson trichrome staining (bar = 150 μm; *n* = 5 per group). **e** *I*/*M* ratio in different groups after EPC transplantation. **f** Neointimal area in different groups after EPC transplantation (*p < 0.05; **p < 0.001)

Saline-treated EPCs (control group), CXCR7-EPCs^Nrf2-WT^ (CXCR7-EPC^Nrf2-WT^ group), or CXCR7-EPCs^Nrf2-KD^ (CXCR7-EPC^Nrf2-KD^ group) were transplanted into the rats with both diabetes and carotid artery injury to determine whether knockdown of Nrf2 impairs CXCR7-EPC-mediated endothelial repair in DM. CXCR7-EPC^Nrf2-WT^ transplantation accelerated reendothelialization (CXCR7-EPC^Nrf2-WT^ vs control group: 7 days, 91.42 ± 10.03% vs 52.19 ± 4.55%, *p* < 0.001; 14 days, 97.05 ± 11.86% vs 89.7 ± 8.16%, *p* = 0.429) and inhibited neointimal hyperplasia (CXCR7-EPC^Nrf2-WT^ vs control group: *I*/*M* ratio, 23.85 ± 5.67% vs 75.63 ± 10.45%, *p* < 0.001; neointimal area, 4.72 ± 1.33 vs 17.75 ± 2.19 × 10^3^ μm^2^, *p* < 0.001) compared with the corresponding parameters in the control group. However, blockade of Nrf2 significantly impaired EPC-mediated endothelial repair. The reendothelialization rate in the CXCR7-EPC^Nrf2-KD^ group was lower than that in the CXCR7-EPC^Nrf2-WT^ group 7 days after treatment (CXCR7-EPC^Nrf2-WT^ vs CXCR7-EPC^Nrf2-KD^: 7 days, 91.42 ± 10.03% vs 61.88 ± 10.15%, *p* < 0.001; 14 days, 97.05 ± 11.86% vs 91.03 ± 10.52%, *p* = 0.53). However, the reendothelialization rate was not significantly different between the CXCR7-EPCs^Nrf2-KD^ and control groups (CXCR7-EPC^Nrf2-KD^ vs control group: 7 days, 61.88 ± 10.15% vs 52.19 ± 4.55%, *p* = 0.426; 14 days, 91.03 ± 10.52% vs 89.7 ± 8.16%, *p* = 0.658) (Fig. 6a–c). In contrast, the *I*/*M* ratio in the CXCR7-EPC^Nrf2-KD^ group was higher than that in the CXCR7-EPC^Nrf2-WT^ group on day 21 (CXCR7-EPC^Nrf2-WT^ vs CXCR7-EPC^Nrf2-KD^, 23.85 ± 5.67% vs 62.92 ± 4.72%, *p* < 0.001), and the neointimal volume in the CXCR7-EPC^Nrf2-KD^ group was also higher than that in the CXCR7-EPC^Nrf2-WT^ group on day 21 (CXCR7-EPC^Nrf2-WT^ vs CXCR7-EPC^Nrf2-KD^, 4.72 ± 1.33 vs 15.86 ± 2.12 × 10^3^ μm^2^, *p* < 0.001) (Fig. 6d, f). Collectively, these findings confirmed that the Keap-1/Nrf2 axis is downstream of the SDF-1/CXCR7 pathway and is involved in regulating EPC function in vivo and in vitro.

## Discussion

Recent developments in the characterization of human peripheral blood mononuclear cell-derived EPCs have led to the identification of two types of EPCs: early EPCs and late outgrowth EPCs [25–28]. The surface antigen molecules most commonly used to immunophenotype EPCs include CD34, CD133, KDR, CD31, CD45, and CD14 [29, 30]. Most previous studies considered early EPCs to be positive for CD14 and CD45 [28, 30] and late outgrowth EPCs to be positive for CD34, CD133, and KDR but negative for CD45 [29, 31]. All of these different EPC subtypes contribute to endothelial repair of vascular injury; late outgrowth EPCs can differentiate into endothelial cells, but early EPCs exert beneficial effects through their proangiogenic paracrine activity [32, 33]. Here, we assessed the association between CXCR7 expression and the repair capacity of late outgrowth EPCs in DM. Furthermore, we explored the potential signaling molecules downstream of the SDF-1/CXCR7 axis that are involved in regulating EPC functional activity. We found that DM impaired the repair capacity of EPCs both in vivo and in vitro, and this impairment was attributed to decreased CXCR7 expression. Furthermore, our results confirmed the critical role of the AKT/Keap-1/Nrf2 axis as the downstream signaling target of SDF-1/CXCR7 in regulating EPC functional activity. Most importantly, we found that transplantation of CXCR7-primed EPCs but not normal or diabetic EPCs was an effective method to promote reendothelialization and inhibit neointimal hyperplasia in DM.

Many studies have shown that DM impairs EPC functions in humans and mice [34, 35]. Specific important molecules, such as CXCR4, NO, and p66Shc, have been identified to control the dysfunction of cultured and circulating putative EPCs [11]. Several recent studies have indicated that the SDF-1/CXCR7 axis is involved in regulating EPC functions, including proliferation, adhesion, angiogenesis, and endothelial repair, both in vitro and in vivo [17–19]. For example, Dai et al. pointed out that DM attenuates CXCR7 expression and impairs the angiogenic function of EPCs [18]. However, the role of the SDF-1/CXCR7 axis in regulating EPC-mediated endothelial repair is still unclear. Our results confirmed that decreasing CXCR7 expression in EPCs contributes to delayed reendothelialization in DM. Furthermore, our in vitro study revealed that the delayed endothelial repair in DM is attributed mainly to the impaired adhesion and repair capacities of EPCs, because elevating CXCR7 expression in EPCs restored their repair capacity both in vitro and in vivo. However, the detailed molecular mechanism by which CXCR7 expression is downregulated in diabetic EPCs remains unclear and needs further exploration.

Heterodimerization of CXCR7 with CXCR4 induces the internalization and degradation of CXCR4 [36], thus interfering with CXCR4-induced Gαi protein-mediated signaling [37, 38]. However, heterodimerization of CXCR7 with CXCR4 favors β-arrestin recruitment [39], stimulating Akt phosphorylation [40] and integrin activation [41]. On the other hand, the CXCL12 scavenging function of CXCR7 reinforces CXCR4-mediated signaling by preventing downregulation of CXCR4 surface expression and function mediated through excessive CXCL12 concentrations [42]. Though the crosstalk between the SDF-1/CXCR4 and SDF-1/CXCR7 axes in regulating EPC function has been well illustrated by previous studies, the downstream mechanism by which the SDF-1/CXCR7 axis is involved in regulating EPC functional activity remains largely unknown. Wang et al. pointed out that Nrf2 protects EPCs against cellular dysfunction by decreasing cellular senescence under hyperglycemic conditions [22]. Moreover, activated Nrf2 can bind to AREs and increase HO-1 and NQO-1 expression, thus exerting a cytoprotective effect via the production of bioactive products or depletion of intracellular sulfhydryl pools [20, 21]. Dai et al. pointed out that elevating CXCR7 expression improves EPC functional activity, including their angiogenic and migratory activity, by stimulating HO-1 and NQO-1 expression [18]. Our results also confirm that the SDF-1/CXCR7 axis regulates EPC functional activity by activating the Keap-1/Nrf2 pathway, because upregulation of CXCR7 stimulated Akt phosphorylation, Keap-1 inactivation, Nrf2 accumulation, and HO-1 and NQO-1 expression, accompanied by enhancement of EPC functional activity, which was reversed by blocking Akt or Nrf2 with LY2900 or shRNA, respectively. Different from the results of Dai et al.’s study, our results confirmed that the SDF-1/CXCR7 axis positively modulates the adhesion and repair capacities but not the angiogenic ability of EPCs by activating Keap-1/Nrf2-associated antioxidant genes, including HO-1 and NQO-1. However, the molecular mechanism by which HO-1 and NQO-1 enhance the adhesion and repair capacities of EPCs remains unclear and needs further study.

Hyperglycemia is an important risk factor for vascular disease and contributes to the development of atherosclerosis and vascular remodeling after vascular injury. Transplantation of exogenous EPCs was evaluated as a potential method to promote endothelial repair by directly elevating the circulating EPC level in a rabbit model and a hypercholesterolemic rat model [43, 44]. However, the role of EPC transplantation in regulating endothelial repair under hyperglycemic conditions remains unclear. In this study, we initially applied transplanted EPCs transfected with the CXCR7 gene to treat vascular injury under hyperglycemic conditions. Unfortunately, our results showed that neither normal EPCs nor diabetic EPCs accelerated endothelial repair in the diabetic rat model. However, the therapeutic effect of CXCR7-primed EPC transplantation on reendothelialization in DM was promising. These results may be partially due to DM-induced impairment of the adhesion and regeneration capacities of EPCs but not of CXCR7-primed EPCs, which was well illustrated in our in vitro study. These results are also well supported by the findings of Dai et al., who showed that upregulating CXCR7 expression abolished the increase in the ROS level in CXCR7-EPCs exposed to ox-LDL or high glucose (HG) by stimulating HO-1 and NQO-1 expression [18]. Collectively, these data suggest that CXCR7 improves the efficiency of EPC transplantation for the treatment of diabetes-associated vascular complications, such as peripheral artery disease, coronary artery disease, vascular restenosis, and thrombosis, after interventional therapy.

## Conclusions

**Fig. 7:**
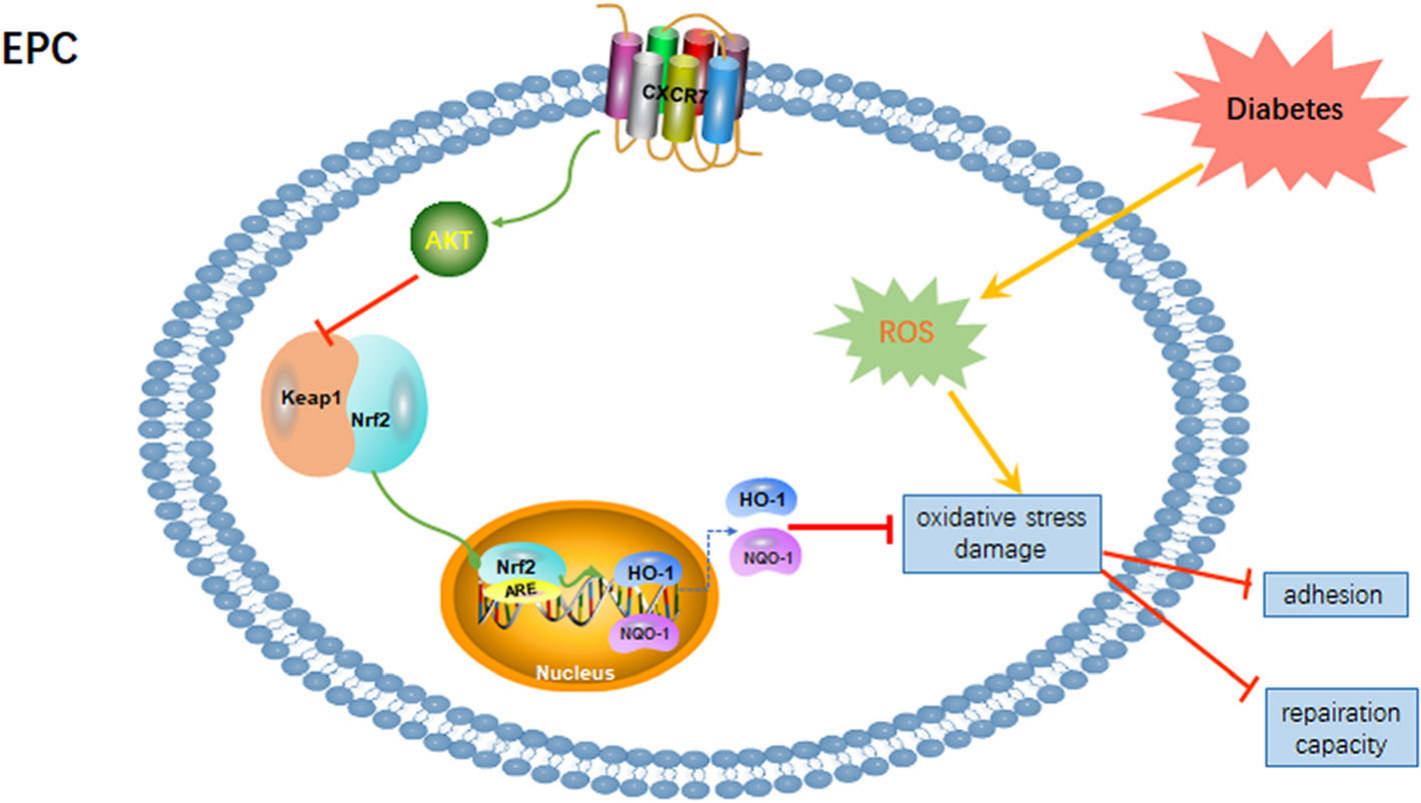
The protective effect of the SDF-1/CXCR7 axis on diabetic EPCs. Diabetes decreased the expression of CXCR7 in diabetic EPCs and impaired the adhesion and repair capacities of EPCs in vitro and in vivo. However, elevating CXCR7 expression enhanced the functional activity of EPCs by activating the Akt-associated Keap-1/Nrf2 axis, counteracting the oxidative stress damage induced by ROS in DM

As shown in Fig. 7, elevating CXCR7 expression preserved the functional activity of diabetic EPCs and protected against diabetes-associated oxidative stress damage by activating the Keap-1/Nrf2 axis in vivo and in vitro. Furthermore, transplantation of CXCR7-EPCs was an effective method to promote reendothelialization, indicating the potential future use of CXCR7-EPCs to treat diabetes-associated vascular disease.

## Supplementary Information

**Additional file 1.**

**Additional file 2.**

**Additional file 3.**

## Data Availability

The datasets used and analyzed in current study are available from the corresponding author based on reasonable request.

## Abbreviations

EPCs: Endothelial progenitor cells
LOCs: Late outgrowth EPCs
CXCR7: C-X-C chemokine receptor type 7
SDF-1: Stromal-Derived Factor-1
Keap-1: Kelch-like ECH-associated protein 1
Nrf2: Nuclear factor erythroid 2-related factor 2
DM: Diabetes mellitus
ARE: Antioxidant response element
HO-1: Heme oxygenase − 1
NQO-1: NADPH quinone oxidoreductase 1
ROS: Reactive oxygen species
GFP: Green fluorescent protein
HUVEC: Human umbilical vein endothelial cell
