# Supplementary figures and images for: Upregulating CXCR7 accelerates endothelial progenitor cell-mediated endothelial repair by activating Akt/Keap-1/Nrf2 signaling in diabetes mellitus
Source: Stem Cell Res Ther. 2021 May 3;12:264. doi: 10.1186/s13287-021-02324-7 (PMC8091720; doi:10.1186/s13287-021-02324-7)

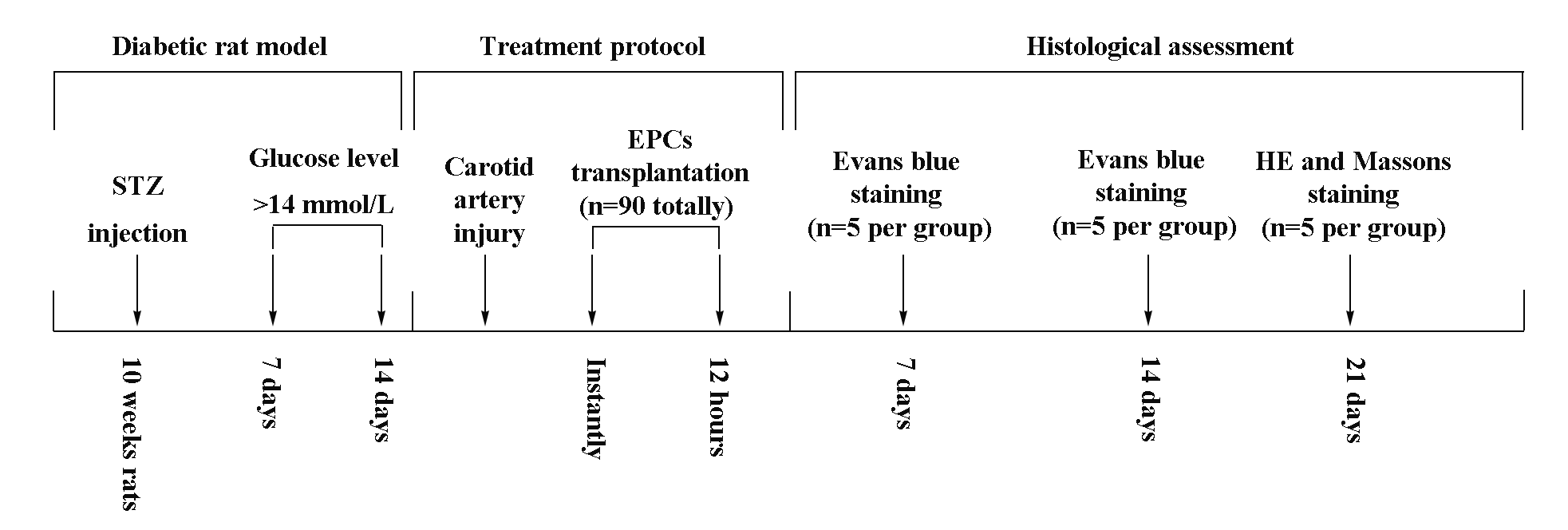

Supplement: Supplementary file 1 — Additional file 1. [file 13287_2021_2324_MOESM1_ESM.tif]

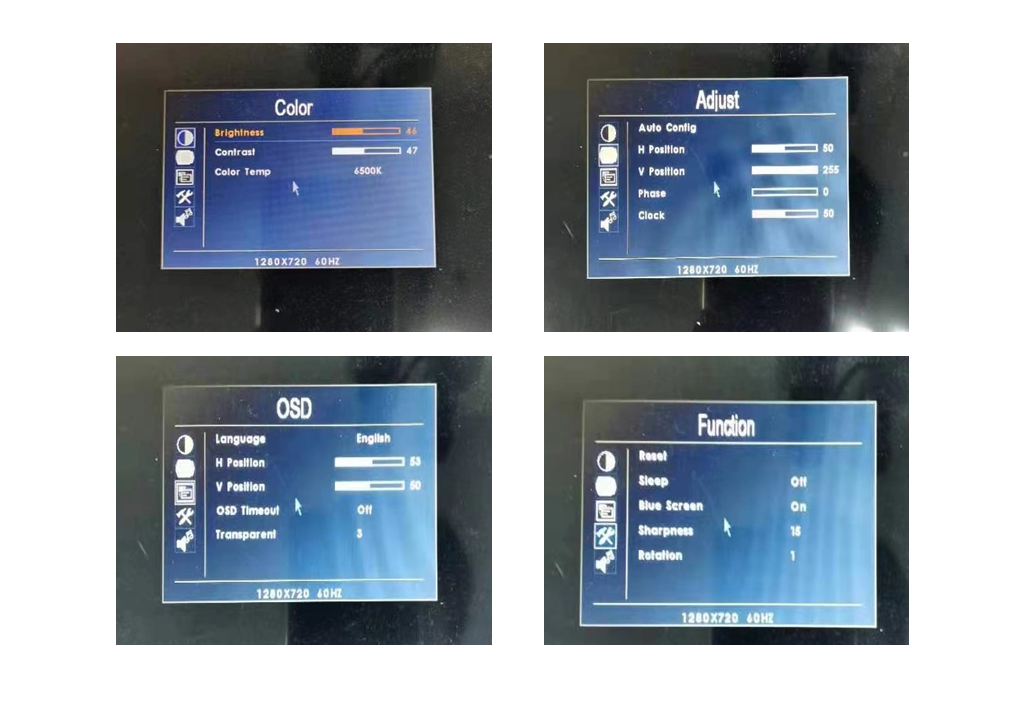

Supplement: Supplementary file 2 — Additional file 2. [file 13287_2021_2324_MOESM2_ESM.tif]

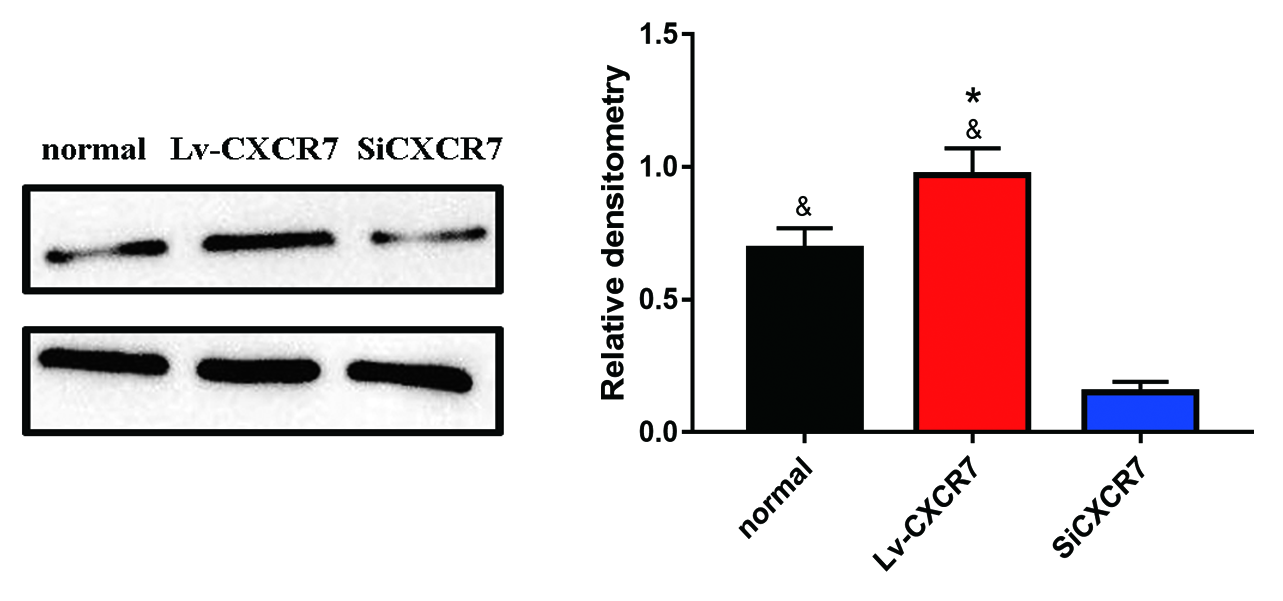

Supplement: Supplementary file 3 — Additional file 3. [file 13287_2021_2324_MOESM3_ESM.tif]
